# Severe Anemia in a Child With Newly Diagnosed Celiac Disease and Suspected Parvovirus B19-Associated Erythroid Suppression

**DOI:** 10.14309/crj.0000000000002249

**Published:** 2026-07-23

**Authors:** Lena Sarhan, Serina B. Beydoun, Bulent Ozgonenel, Ali G. Saad, Sara Evans, Tony Lulgjuraj

**Affiliations:** 1 Department of Pediatrics, Children's Hospital of Michigan, Detroit, MI; 2 Department of Pediatric Gastroenterology, Children's Hospital of Michigan, Detroit, MI; 3 Department of Pediatric Hematology-Oncology, Children's Hospital of Michigan, Detroit, MI; 4 Department of Pathology, School of Medicine, Wayne State University, Detroit, MI; 5 Bloom Pediatrics, Birmingham, MI

**Keywords:** celiac disease, anemia, infection

## Abstract

Celiac disease (CeD) is an autoimmune disorder triggered by gluten ingestion. While anemia is common due to nutrient malabsorption or chronic inflammation, severe anemia is rare. We present a case of a 5-year-old boy with newly diagnosed CeD who developed severe anemia due to parvovirus B19 (PB19) infection associated with pure red cell aplasia resulting in hospitalization. Evaluation revealed positive PB19 polymerase chain reaction and findings consistent with PB19-pure red cell aplasia. The prolonged infection was likely exacerbated by nutritional and immune compromise from untreated CeD. This case highlights the importance of considering causes of severe anemia beyond nutritional deficiencies in children with CeD.

## INTRODUCTION

Celiac disease (CeD) is an autoimmune condition triggered by the ingestion of gluten in genetically predisposed individuals, leading to small intestinal villous atrophy.^1^ The classic presentation includes symptoms such as diarrhea, abdominal pain, bloating, and failure to thrive.^2^ Anemia may result from the malabsorption of key nutrients, such as iron, and from anemia of chronic disease. However, severe anemia is rare. Parvovirus B19 (PB19) infection can cause a pure red cell aplasia (PRCA), leading to severe, transfusion-dependent anemia in immunocompromised patients, but is rare in immunocompetent patients.^3^ We present a case of progressive, severe anemia in a patient with CeD that we suspect was caused by a PB19-associated PRCA.

## CASE REPORT

**Figure 1.**
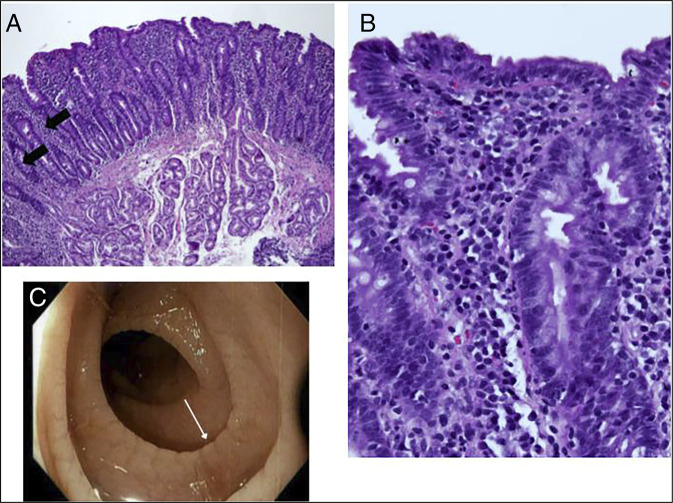
Endoscopic evaluation for celiac disease: (A) Histologic examination shows near total effacement of the villous architecture. There is prominent crypt hyperplasia (black arrows). Lamina propria cellularity is significantly increased. (B) Higher magnification (200×) showing increased lamina propria cellularity and increased intraepithelial lymphocytes (white arrows). The constellation of findings is that of Marsh stage 3b. (C) Endoscopic imaging of duodenum, demonstrating scalloping (white arrow), findings consistent with celiac disease.

A 5-year-old boy underwent esophagogastroduodenoscopy (EGD) for evaluation of suspected CeD. Routine testing on the day of the procedure revealed severe anemia with hemoglobin 5.6 g/dL (mean corpuscular volume 81 fL); repeat testing confirmed hemoglobin 4.0 g/dL. EGD demonstrated duodenal scalloping (Figure 1).

Seven weeks prior, he was evaluated for fatigue and poor weight gain by his pediatrician. Testing then showed markedly elevated tissue transglutaminase IgA (>250 mg/dL) with normal total IgA (172 mg/dL). Mild normocytic anemia was present (hemoglobin 10.2 g/dL, mean corpuscular volume [MCV] 82 fL). Over the subsequent 4–6 weeks, he developed worsening fatigue and a “slapped cheek” facial rash.

Following EGD, he was admitted for packed red blood cell (RBC) transfusion. On examination, he appeared pale and tachycardic but normotensive. There was no evidence of gastrointestinal bleeding and no family history of hematologic disease.

Laboratory evaluation demonstrated normocytic anemia with ferritin 30 ng/mL, serum iron 37 µg/dL, total iron-binding capacity 290 µg/dL, and iron saturation 13%. Reticulocyte count was elevated at 182,000/µL (9.0%). Haptoglobin, lactate dehydrogenase, folate, vitamin B12, thyroid function tests, hemoglobin electrophoresis, eosin-5-maleimide testing, and flow cytometry were unremarkable. Peripheral blood smear showed mostly normocytic, normochromic RBCs with a small proportion of RBCs that were hypochromic, microcytic. A next-generation sequencing panel for congenital anemias identified no pathogenic variants. Epstein-Barr virus and cytomegalovirus polymerase chain reaction were negative. PB19 polymerase chain reaction was positive (2.4 IU/mL).

**Figure 2.**
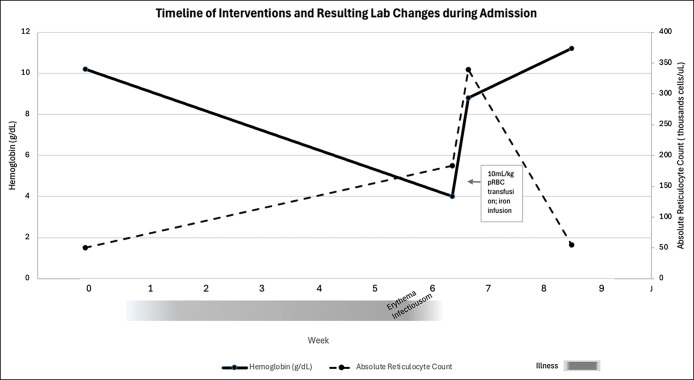
Showing the timeline of events, interventions, and accompanying laboratory values during the patient's course of illness and admission. pRBC, packed red blood cell.

He received packed RBC (10 mL/kg) and intravenous ferric gluconate (1.5 mg/kg), with improvement in hemoglobin and energy (Figure 2). Duodenal biopsies demonstrated Marsh 3 histology, confirming CD (Figure 1). He was discharged on oral iron supplementation and a strict gluten-free diet. At follow-up, we concluded that his severe anemia was most likely attributable to prolonged PB19-infection leading to PRCA superimposed on preexisting anemia of chronic disease from CD.

## DISCUSSION

Hematologic manifestations of CeD are common, most frequently iron deficiency anemia from malabsorption. However, red cell aplasia is rarely described. While aplastic anemia in CeD has been attributed to autoreactive T-cell-mediated destruction, clinically significant PB19-associated PRCA has not been previously reported in a patient with CeD.^3^

PB19 infects erythroid progenitor cells by binding to the P antigen, causing temporary arrest of erythropoiesis.^4^ In healthy individuals, this results in transient reticulocytopenia lasting 7–10 days with a modest hemoglobin decline of approximately 1 g/dL. Rash and arthralgia typically appear as reticulocyte counts recover and anti-PB19 IgM antibodies develop, leading to viral clearance.^4^ Patients with hemolytic disorders may develop transient aplastic crisis with severe anemia, while immunocompromised individuals with persistent infection beyond 8 weeks may develop chronic PRCA requiring transfusion support.^5–8^

Our patient experienced a hemoglobin decline of approximately 6 g/dL—far exceeding that expected in immunocompetent hosts. This occurred in the setting of preexisting anemia, with a baseline hemoglobin of 10.2 g/dL—which likely reflects combined iron deficiency and anemia of chronic disease from untreated CeD. This reduced hematologic reserve lowered the threshold for symptomatic anemia requiring transfusion. In the setting of a compatible viral prodrome and detectable PB19 viremia, this presentation is best explained by PB19-driven erythroid suppression superimposed on a compromised baseline (Figure 2).

This presentation does not meet criteria for transient aplastic crisis, as there was no underlying hemolytic disorder, nor for chronic PRCA, defined as a persistent viremia beyond 8 weeks. Instead, the duration and severity of anemia suggest an intermediate-duration erythroid suppression best described as presumed prolonged PB19 infection causing PRCA.

Interpretation of the iron studies is complicated by a mixed process. PB19-associated hypoproliferation likely drove the hemoglobin decline, while concurrent anemia of chronic inflammation from active CeD contributed to impaired iron utilization and a low-normal ferritin. Mild iron deficiency from malabsorption and increased iron demand during recovery likely further reduced serum iron and transferrin saturation. The normal MCV and predominantly normocytic smear support a mixed etiology rather than isolated iron deficiency. The absence of an elevated ferritin despite inflammation may reflect concurrent iron deficiency blunting its rise as an acute-phase reactant.

Establishing a precise timeline remains challenging, as much of the course relied on caregiver-reported symptoms. The low PB19 DNA titer and elevated reticulocyte count at admission suggest evaluation during early marrow recovery, aided by transfusion and intravenous iron. Despite these limitations, the magnitude of hemoglobin decline, viral prodrome, detectable viremia, and subsequent recovery are most consistent with PB19-associated PRCA secondary to prolonged infection.

The prolonged infection raises the possibility of functional immunodeficiency related to untreated CeD. Chronic intestinal inflammation and micronutrient deficiencies may impair immune function and delay viral clearance in addition to contributing to iron deficiency anemia, folate, and B12 deficiency, which can lead to hematologic complications such as anemia.^9^ Although he was not classically immunocompromised, underlying CeD may have predisposed him to a prolonged PB19 infection leading to an exaggerated hemoglobin decline, although this remains speculative.

This case highlights an unusual presentation of severe PB19-associated PRCA in a child with newly diagnosed CeD and underscores the complex causes of anemia in this population. Although micronutrient deficiency is common, severe anemia should prompt consideration of additional processes, including viral-mediated erythroid suppression. Overlapping mechanisms—viral suppression, chronic inflammation, and mild iron deficiency—can create a complex presentation, emphasizing the importance of maintaining a broad differential diagnosis even in patients without overt immunocompromise.

## DISCLOSURES

Author contributions: L. Sarhan wrote and edited the manuscript. S. Beydoun wrote and edited the manuscript. B. Ozgonenel wrote and edited the manuscript. AG Saad provided pathology images and edited the manuscript. S. Evans edited the manuscript. T. Lulgjuraj wrote and edited the manuscript. T. Lulgjuraj is the article guarantor.

Acknowledgments: An artificial intelligence tool (ChatGPT, OpenAI, San Francisco, CA) was used for language refinement and editing support during manuscript preparation. The authors affirm full responsibility for the manuscript's content, including sections refined with artificial intelligence assistance.

Financial disclosure: None to report.

Informed consent was obtained for this case report.

## ABBREVIATIONS:

CD: celiac disease
CMV: cytomegalovirus
EBV: Epstein–Barr virus
EGD: esophagogastroduodenoscopy
IgA: immunoglobulin A
IgM: immunoglobulin M
MCV: mean corpuscular volume
PB19: Parvovirus B19
PCR: polymerase chain reaction
pRBC: packed red blood cells
PRCA: pure red cell aplasia
RBC: red blood cell(s)
